# Mean platelet volume is more important than age for defining reference intervals of platelet counts

**DOI:** 10.1371/journal.pone.0213658

**Published:** 2019-03-14

**Authors:** Till Ittermann, Martin A. Feig, Astrid Petersmann, Dörte Radke, Andreas Greinacher, Henry Völzke, Thomas Thiele

**Affiliations:** 1 Institute for Community Medicine, University Medicine Greifswald, Greifswald, Germany; 2 Institute for Immunology and Transfusion Medicine, University Medicine Greifswald, Greifswald, Germany; 3 Institute for Clinical Chemistry and Laboratory Medicine; University Medicine Greifswald, Greifswald, Germany; Oregon State University, UNITED STATES

## Abstract

**Background:**

Platelet count is known to be associated with sex, age and mean platelet volume (MPV). Sex and age were proposed for adjustment of platelet count reference intervals, but MPV is currently not used for further adjustment. We investigated the association of MPV, age and sex with platelet counts and established individualized reference ranges respecting MPV.

**Methods:**

The association of platelet count with age, sex and MPV was assessed in healthy participants (n = 3,033 individuals; 1,542 women) in the cross-sectional population-based cohort Study of Health in Pomerania. Reference intervals respecting age, sex, and MPV were estimated using quantile regressions for the 2.5^th^ and 97.5^th^ percentile.

**Results:**

Women had higher platelet counts than men (239 vs. 207 x10^9^/L, p<0.001). Platelet counts correlated with age (p<0.001) and MPV (p<0.001). Quantile regression of lower and upper platelet count limits correlated less with age in female (p = 0.047 for 2.5^th^ percentile; p = 0.906 for 97.5^th^ percentile) and male subjects (p = 0.029 for 2.5^th^ percentile; p = 0.195 for 97.5^th^ percentile) compared to MPV (p<0.001 for upper and lower limit for both sexes). After adjustment for MPV, age did no longer correlate with the 2.5^th^ (p = 0.165) or 97.5^th^ percentile (p = 0.999) of platelet count. In contrast, after adjustment for age, MPV levels still significantly correlated with 2.5^th^, 50^th^ and 97.5^th^ percentile (p<0.001).

**Conclusion:**

MPV and sex have a stronger association with platelet count than age. MPV should be considered to adjust platelet count reference intervals and needs to be respected as confounder for platelet counts in epidemiological studies and clinical practice.

## Introduction

Platelets play a key role in primary hemostasis, promote angiogenesis [1] and mediate immune defense [2]. The platelet count is genetically determined [3–6] and a range between 150 and 450 x10^9^ platelets/L [7] is widely accepted as the physiological range for platelet counts. In the absence of disease, the individual platelet count is highly stable, but may decline with age [8].

Sex [9, 10], ethnicity [11, 12], environmental conditions [5, 13], and seasonal differences are known to influence platelet counts [4]. Hence, the concept of adjusted platelet counts has been proposed in order to reduce misdiagnoses of thrombocytopenia. Including 12,142 healthy subjects, Segal and Moliterno adjusted mean platelet counts for ethnicity, age, alcohol use, nutritional and inflammatory covariates separately for men and for women [14]. Similarly Biino et al. suggested age-, sex- and ethnicity-adjusted reference intervals based on 12,517 healthy individuals [3].

Another platelet parameter shown to correlate with platelet counts is the mean platelet volume (MPV). There is a strong inverse correlation between platelet count and platelet size, as measured by MPV [7, 15–17], even though this correlation is nonlinear [13, 16]. MPV is also genetically determined [18–20] and neither affected by gender [21, 22] nor life-style [23], while the influence of age on MPV is debated. Some authors consider MPV to be independent of age [24, 25], while others found an association between age and increased MPV in mice [26] and humans [15]. Up to now, MPV has not been considered as a parameter to be included in the estimation of reference ranges of the platelet count, which may be due to the fact, that MPV measurement is not well standardized and device dependent [27].

Based on the population-based Study of Health in Pomerania (SHIP) [28], we investigated the association of platelet count with sex, age and MPV. We provide evidence that MPV is more important to be included in platelet count reference value estimation than age. We present new reference values for platelet counts adjusted for sex and MPV for a Middle-European population for the Sysmex platform. These data may help to redefine reference ranges for platelet counts in order to avoid over- and under-diagnosis of thrombocytopenia and thrombocytosis. More importantly, we indicate that MPV needs to be considered as a confounder in epidemiological studies, which include platelet counts.

## Methods

### Study population

SHIP-TREND-0 is a population-based cross-sectional study conducted in the Northeast of Germany between 2008 and 2012 [28]. For SHIP-TREND-0 a random, age- and sex-stratified sample of 8,826 eligible subjects was drawn from the population registry of which 4,420 subjects participated (net response 50.1%). All participants gave informed written consent and SHIP-TREND-0 followed the recommendations of the Declaration of Helsinki and was approved by the Ethics Committee of the University of Greifswald.

Of the 4,420 individuals, 29 had missing data in platelet count or MPV. Between August 30^th^ 2011 and March 29^th^ 2012 platelet count and MPV measurements were conducted on a different analysis platform than during the rest of the study. The data from 479 participants, who were examined during this time period, were not used for the present analysis. These participants did not differ significantly regarding age, sex, body mass index and smoking status from the participants included in our analyses. To define a healthy reference population we excluded 265 participants who stated to have had cancer during their lifetime in a personal interview or were on cancer treatment. Since previous studies demonstrated a significant impact of inflammation on MPV [29], we excluded individuals with increased (>4 g/L, n = 361) or decreased (< 1.8 g/L, n = 43) plasma fibrinogen levels (based on reference levels used in the central laboratory of the University Medicine Greifswald) and individuals with increased (> 10.7 x 10^9^/L, n = 45) or decreased (< 3.8 x 10^9^/L, n = 165) white blood cell count [30] resulting in a reference population of 3,033 individuals (1,542 women, S1 Fig).

### Measurements

Fasting blood samples were collected between 7 a.m. and 1 p.m. from the cubital vein in the supine position. Aliquots were prepared for immediate analysis and for storage at -80°C. Blood samples were analyzed at the central laboratory of the University Medicine Greifswald. Platelet count and MPV were determined at maximum two hours after sample taking in EDTA anticoagulated whole blood on the Sysmex platform XE 5000 (Sysmex Corporation, Kobe, Japan). The analyzer was calibrated according to the manufacturer’s instructions and quality control was performed internally as well as externally, the latter by regular participation in national external quality assessment schemes (EQAS). The inter-assay coefficients of variation for platelet count were 5.66%, 4.29% and 3.67% for low, medium and high concentrations of control material.

### Statistical analyses

To correlate platelet count and MPV with age as well as platelet count with MPV we used Spearman’s rank correlation coefficients.

As recommended by the Clinical and Laboratory Standards Institute (CLSI) we used a non-parametric method to establish reference intervals for platelet counts [31]. We used quantile regression to directly model percentiles of platelet count in dependence of MPV, age, and sex [32]. Reference intervals were defined according to the 2.5^th^ and 97.5^th^ percentile of platelet counts. To account for possible non-linear associations between MPV or age with the respective percentile of platelet count we applied multivariable fractional polynomials [33]. This method tests transformations of the continuous exposure variables (MPV and age) by comparing the fit of quantile regression models with and without transformations of the exposure variables. If the fit of the model with the transformed exposure variable was superior to the fit of the model without exposure transformation, the exposure variable used was transformed in the quantile regression model. A p<0.05 was considered as statistically significant. All analyses were performed with Stata 12.1 (Stata Corporation, College Station, TX, USA).

## Results

### Study population

Median age in the reference population was 51 years (25^th^ percentile 39 years, 75^th^ percentile 62 years). Median age differed significantly between males (52 years) and females (50 years; p = 0.008). The reference population was similarly distributed over the age groups between 18 to 80 years (S2 Fig). Median platelet count was 222 x10^9^/L (25^th^ percentile 190x10^9^/L and 75^th^ percentile 259x10^9^/L) and median MPV was 10.3 fL (25^th^ percentile 9.8 fL and 75^th^ percentile 10.9 fL) in the reference population. In our study population, 2,003 individuals took at least one medication. Median platelet count (221 x10^9^/L in individuals with medication and 223 x10^9^/L in individuals without) and MPV (10.3 fL in both groups) were similar across individuals with and without medication intake.

### Association of platelet counts with sex, age and MPV

**Fig 1:**
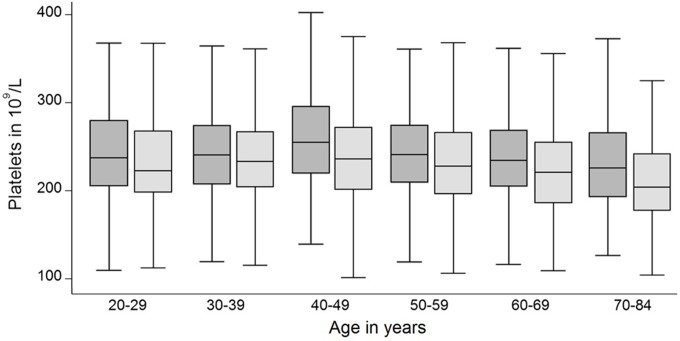
Box plots of platelet count for females (dark) and males (light) stratified by age. Median platelet count is significantly higher in females (p<0.001 in all age strata). Platelet count correlated significantly with age.

**Fig 2:**
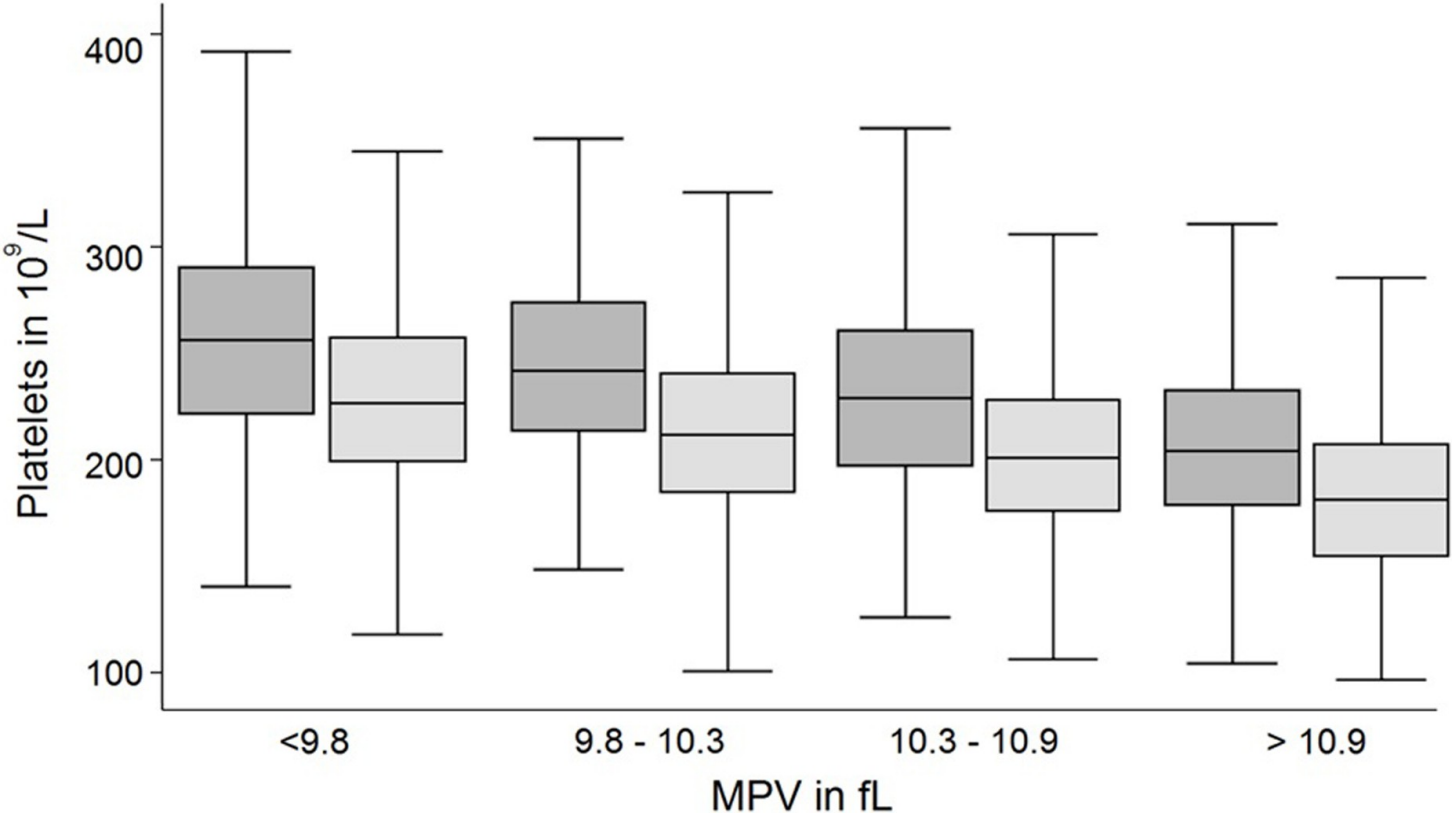
Box plots of platelet count for females (dark) and males (light) stratified by MPV. Median MPV inversely correlates with platelet counts in females and males (Spearman’s rho = -0.3735; p<0.0001).

**Fig 3:**
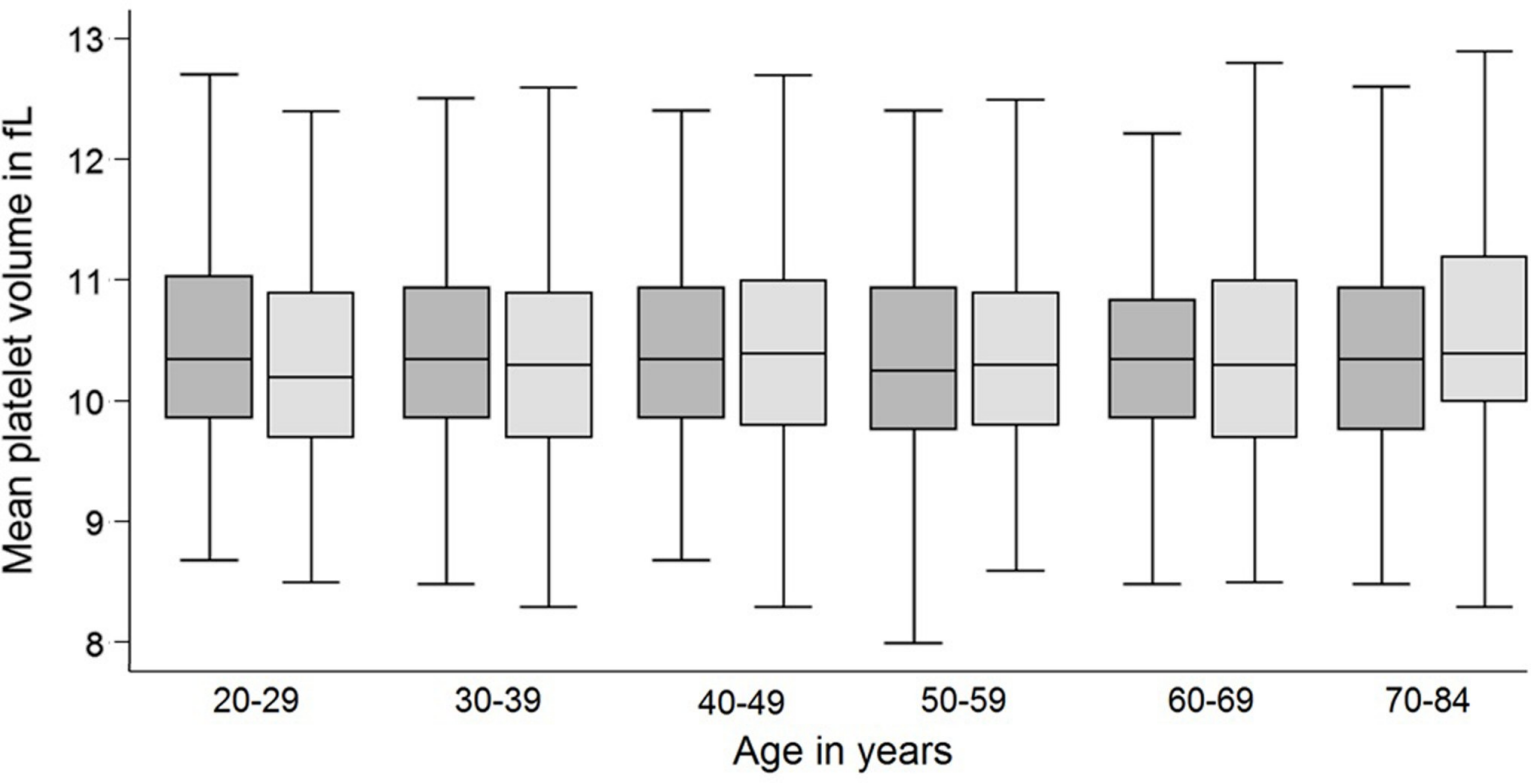
Box plots of MPV for females (dark) and males (light) stratified by age. Median MPV is similar between males and females and does not correlate with age.

Platelet counts were associated with all three examined parameters: sex, age and MPV. Median platelet counts were higher in females than in males (239 x10^9^/L vs. 207 x10^9^/L; p<0.001) which was observed in all age-groups (Fig 1). Platelet count correlated significantly with age (Spearman’s rho = 0.4950; p<0.0001). Moreover, platelet counts inversely correlated with MPV (Spearman’s rho = -0.3735; p<0.0001; Fig 2). In contrast, median MPV was similar between males and females (10.3 fl vs. 10.3 fL, p = 0.372) and did not correlate with age (Fig 3).

### Correlation of platelet count percentiles with age and MPV

The correlation of platelet count reference intervals with age and MPV was determined by means of quantile regression for the 2.5^th^ and the 97.5^th^ percentiles (Table 1). Not all percentiles of the platelet counts correlated with age. The 2.5^th^ percentile correlated with age in males (p = 0.029) and females (p = 0.047), whereas the 97.5^th^ percentile of platelet counts did not correlate with age (for males: p = 0.195; for females: p = 0.906).

**Table 1:** Formulas for 2.5^th^, 50^th^ and 97.5^th^ percentile of platelet count unadjusted and adjusted for age based on data from 1,491 males and 1,542 females.

Sex	Percentile	Formula for platelet count percentiles*	p* MPV	p* age
**Unadjusted for age**				
**Males** **(n = 1,491)**	2.5^th^	372.0385–22.6923*MPV	<0.001	
	50^th^	428.6522–21.3044*MPV	<0.001	
	97.5^th^	556.7777–24.4444*MPV	<0.001	
**Females (n = 1,542)**	2.5^th^	291.8572–12.8572*MPV	<0.001	
	50^th^	496.8824–24.7059*MPV	<0.001	
	97.5^th^	654.9999–29.9999*MPV	<0.001	
**Age-adjusted**				
**Males** **(n = 1,491)**	2.5^th^	389.6208–21.6905*MPV– 0.4821*age	<0.001	0.029
	50^th^	438.9172–21.2408*MPV– 0.0614*(age/10)^3^	<0.001	<0.001
	97.5^th^	592.2213–25.7482*MPV– 197.5671*1/(age/10)^2^–0.0717+(age/10)^3^	<0.001	0.195
**Females (n = 1,542)**	2.5^th^	322.2372–14.2500*MPV– 0.2875*age	<0.001	0.047
	50^th^	512.1741–24.3939*MPV– 0.3864*age	<0.001	<0.001
	97.5^th^	643.5572–29.2135*MPV + 0.0539*age	<0.001	0.906

*Formulas and p-values taken from quantile regression for the respective percentile. MPV = mean platelet volume.

After adjustment for MPV, no significant correlation between the 2.5^th^ (p = 0.165) or 97.5^th^ percentile (p = 0.999) of platelet counts and age was observed. This was confirmed in males and females.

In males and females, platelet counts decreased significantly with increasing MPV over all percentiles (p<0.001). After adjustment for age, the 2.5^th^ and 97.5^th^ percentiles of the platelet counts still correlated with MPV in males and females (p<0.001 for each comparison).

### Proposed reference values adjusted for sex, age and MPV for the Sysmex platform

In dependency of MPV, age, and sex, adjusted reference ranges for platelet counts can be determined. If adjusted for age, reference platelet count for females is between (322.2372–14.2500*MPV– 0.2875*age) x10^9^/L and (643.5572–29.2135*MPV + 0.0539*age) x10^9^/L. For males the reference range for platelet count can be given as (389.6208–21.6905*MPV– 0.4821*age) x10^9^/L for the lower limit and (592.2213–25.7482*MPV– 197.5671*1/(age/10)^2^–0.0717+(age/10)^3^) x10^9^/L for the upper limit.

**Fig 4:**
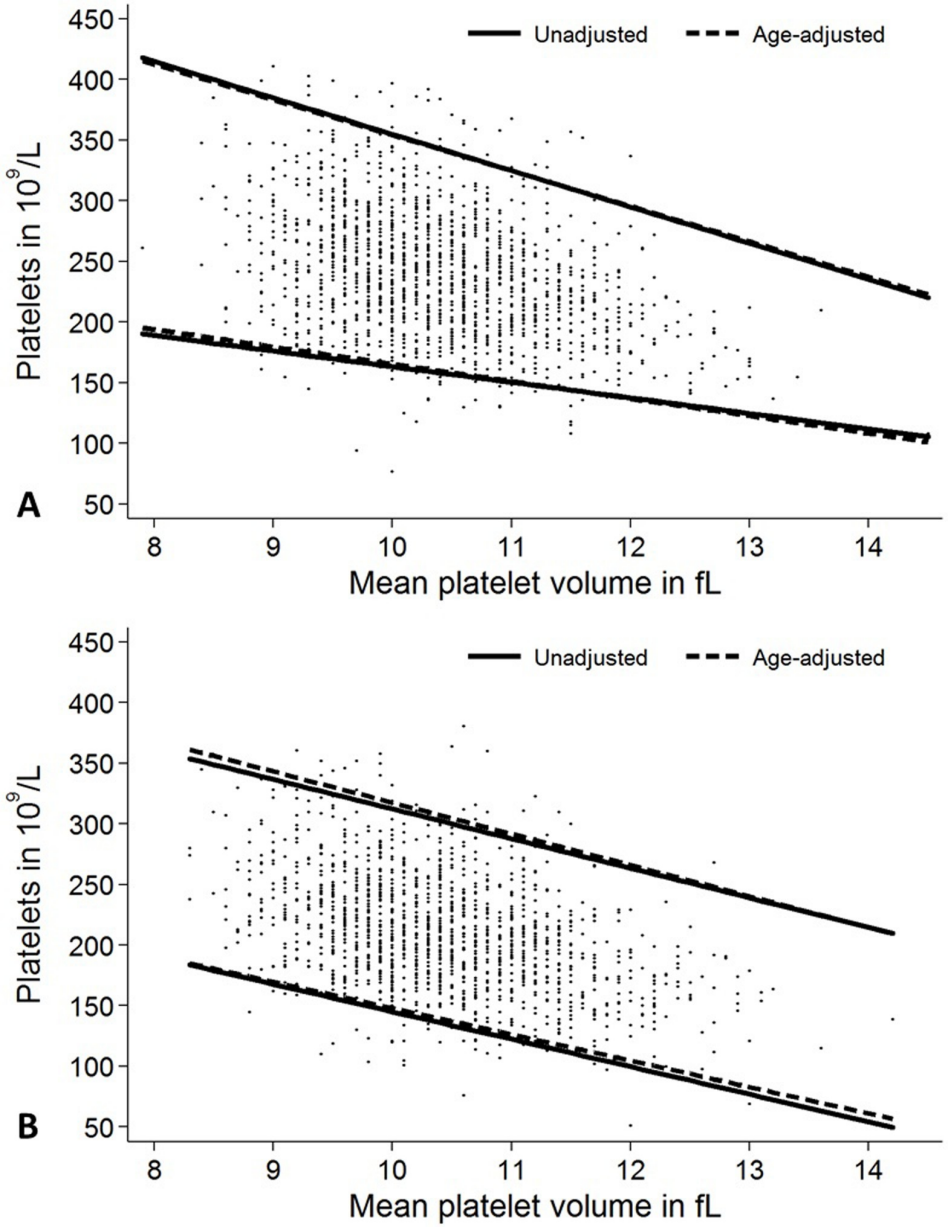
**Reference intervals age-adjusted and unadjusted for age for platelet count in dependence of mean platelet volume for (A) females and (B) males.** Adjustment for age has a minor impact on reference values for platelet count in females and males, when MPV is considered for adjustment.

We then compared the reference intervals in dependence of sex and MPV with those obtained by additional age-adjustment (Table 1 and Fig 4). This analysis revealed that the inclusion of age as a contributing factor for the range of platelet counts provides only minimal additional information. MPV has a bigger effect size for the estimation of reference intervals for platelet counts than age. Therefore the estimation of reference range limits could be simplified by ignoring age for adjustment of platelet counts (291.8572–12.8572*MPV) x10^9^/L and (654.9999–29.9999*MPV) x10^9^/L for females and between (372.0385–22.6923*MPV) x10^9^/L and (556.7777–24.4444*MPV) x10^9^/L for males.

We calculated reference ranges for males and females for different MPVs determined on the Sysmex platform without age-adjustment (Table 2) and propose these as new reference ranges for platelet counts. S1 and S2 Tables show platelet count reference values for females and males for different MPVs and adjusted for age revealing a minor impact of additional age adjustment.

**Table 2:** Pre-estimated reference ranges for platelet count in 10^9^/L in healthy females and males for given MPV.

MPV / Sex	Females	Males
**8 fL**	189–415	191–361
**8.5 fL**	183–400	179–349
**9 fL**	176–385	168–337
**9.5 fL**	170–370	156–325
**10 fL**	163–355	145–312
**10.5 fL**	157–340	134–300
**11 fL**	150–325	122–288
**11.5 fL**	144–310	111–276
**12 fL**	138–295	100–263
**12.5 fL**	131–280	88–251
**13 fL**	125–265	77–239

## Discussion

In this study we confirm that platelet counts are associated with age, sex and MPV and provide evidence that, from these factors, sex and MPV have the strongest association with platelet count reference ranges. In contrast, the impact of age seems to be of minor relevance.

We observed the known association of sex and age as well as MPV on the platelet count [7, 8, 13, 14, 16, 34–37] (Figs 1 and 2). The known inverse relationship between MPV and platelet count was stable across ages and genders in our study and was comparable to other studies [23, 38]. On the other hand, MPV was stable over different ages and between sexes (Fig 3). We extent previous findings by the observation, that MPV correlates stronger with the reference values of platelet counts than age (Table 1). We therefore challenge the concept of adjustment of platelet count reference values for age and sex without respecting MPV. Based on our data, age may even be negligible for the estimation of reference values of platelet counts (Fig 4).

The association of age and sex with platelet counts led to the concept of adjusted reverence values. Age dependent reference values were earlier proposed by Flegar-Mestric et al. [39], who showed that young subjects aged 8–19 years had platelet counts between 178–420 x10^9^/L and those aged 20–70 years had platelet counts between 158–424 x10^9^/L. Adjustment for sex only revealed different platelet count reference ranges in females compared to males (137–347 vs. 144–328 x10^9^/L) [22]. Hong et al. also proposed different reference ranges of 122–334 for females and 111–305 x10^9^/L for males [40].

Adjustment for the combination of age and sex was previously proposed by Biino et al. [8] resulting in a maximal difference of 30–50 x10^9^/L to the widely used reference values. However, when we considered MPV for adjustment of reference ranges, this resulted in considerable differences of the reference platelet count range reaching differences which may be of high clinical relevance for the diagnosis of thrombocytopenia and thrombocytosis (Table 2). For an MPV of 9 fL for a middle aged woman (e.g. 50 years) the proposed reference range is 180–383 x10^9^/L (median = 273 x10^9^/L). At the same age for an MPV of 12 fL the reference interval is estimated 137–296 x10^9^/L. Therefore, considering MPV, the difference in this example would be as big as 43 x10^9^/L for the 2.5^th^ percentile and 87 x10^9^/L for the 97.5^th^ percentile, respectively.

Adjustment of the reference ranges for platelet counts has an impact on clinical decisions. In a retrospective study, personalized reference intervals for platelet counts, respecting age and sex for adjustment, reduced the number of subjects with thrombocytopenia by 44.8% [41], but the number of subjects with thrombocytosis increased. The first prospective study investigating the impact of adjusted reference ranges for platelet counts showed an increased risk of mortality in patients with thrombocytopenia according to the adjusted reference range when compared to patients with thrombocytopenia according to the standard reference range [42]. Based on our study, we propose that sex- and MPV-adjusted reference values for platelet counts may improve the diagnosis of thrombocytopenia and thrombocytosis and should be tested in prospective studies.

The strength of our study is its cross-sectional observation in a large cohort of the general adult population, which shows similar trends compared to previous studies. Limitations are that only healthy adults at the age of 18–85 had been included. The reference values proposed here may therefore not be valid in children. Furthermore, neither environmental nor genetic factors, nor hemoglobin levels were taken into account in this study. Noteworthy, the determination of MPV is not standardized and device dependent [27, 43, 44]. Thus, our results are restricted to measurements performed on the widely used Sysmex platform.

In summary, we show that an adjustment for MPV considerably changes reference values for the platelet count, while age had only a minor effect. Because of its relevance, MPV needs to be respected as a major confounder for platelet counts in epidemiological studies. Finally, we propose to test the utility of sex- and MPV-adjusted reference values for platelet counts for diagnosis of thrombocytopenia and thrombocytosis in upcoming prospective studies.

## Supporting information

S1 Table Pre-estimated reference ranges for platelet count in 10^9^/L in healthy females for given age and MPV. (DOC)

S2 Table Pre-estimated reference ranges for platelet count in 10^9^/L in healthy males for given age and MPV. (DOCX)

S1 Fig Study profile of enrolled and assessed subjects. (TIF)

S2 Fig Histogram of age distribution of study cohort. (TIF)
