# Supplementary material for: Mean platelet volume is more important than age for defining reference intervals of platelet counts
Source: PLoS One. 2019 Mar 14;14(3):e0213658. doi: 10.1371/journal.pone.0213658 (PMC6417788; doi:10.1371/journal.pone.0213658)
Supplement: S1 Table — (DOC) [file pone.0213658.s001.doc]

**Supporting Table 1: Pre-estimated reference ranges for platelet count in 10^9^/L** in healthy females for given age and MPV.

| **MPV \ Age** | **20** | **40** | **60** | **80** |
| --- | --- | --- | --- | --- |
| **8 fL** | 202 - 411 | 197 - 412 | 191 - 413 | 185 - 414 |
| **8.5 fL** | 195 - 396 | 190 - 397 | 184 - 398 | 178 - 400 |
| **9 fL** | 188 - 382 | 182 - 383 | 177 - 384 | 171 - 385 |
| **9.5 fL** | 181 - 367 | 175 - 368 | 170 - 369 | 164 - 370 |
| **10 fL** | 174 - 353 | 168 - 354 | 162 - 355 | 157 - 356 |
| **10.5 fL** | 167 - 338 | 161 - 339 | 155 - 340 | 150 - 341 |
| **11 fL** | 160 - 323 | 154 - 324 | 148 - 325 | 142 - 327 |
| **11.5 fL** | 153 - 309 | 147 - 310 | 141 - 311 | 135 - 312 |
| **12 fL** | 145 - 294 | 140 - 295 | 134 - 296 | 128 - 297 |
| **12.5 fL** | 138 - 279 | 133 - 281 | 127 - 282 | 121 - 283 |
| **13 fL** | 131 - 265 | 125 - 266 | 120 - 267 | 114 - 268 |
