# Supplementary material for: Mean platelet volume is more important than age for defining reference intervals of platelet counts
Source: PLoS One. 2019 Mar 14;14(3):e0213658. doi: 10.1371/journal.pone.0213658 (PMC6417788; doi:10.1371/journal.pone.0213658)
Supplement: S2 Table — (DOCX) [file pone.0213658.s002.docx]

**Supporting Table 2: Pre-estimated reference ranges for platelet count in 10^9^/L in healthy males for given age and MPV.**

| **MPV \ Age** | **20** | **40** | **60** | **80** |
| --- | --- | --- | --- | --- |
| **8 fL** | 206 - 336 | 197 - 369 | 187 - 365 | 178 - 346 |
| **8.5 fL** | 196 - 323 | 186 - 356 | 176 - 352 | 167 - 334 |
| **9 fL** | 185 - 311 | 175 - 344 | 165 - 340 | 156 - 321 |
| **9.5 fL** | 174 - 298 | 164 - 331 | 155 - 327 | 145 - 308 |
| **10 fL** | 163 - 285 | 153 - 318 | 144 - 314 | 134 - 295 |
| **10.5 fL** | 152 - 272 | 143 - 305 | 133 - 301 | 123 - 282 |
| **11 fL** | 141 - 259 | 132 - 292 | 122 - 288 | 112 - 269 |
| **11.5 fL** | 131 - 246 | 121 - 279 | 111 - 275 | 102 - 256 |
| **12 fL** | 120 - 233 | 110 - 266 | 100 - 262 | 91 - 243 |
| **12.5 fL** | 109 - 220 | 99 - 253 | 90 - 249 | 80 - 231 |
| **13 fL** | 98 - 208 | 88 - 241 | 79 - 237 | 69 - 218 |
