# Supplementary figures and images for: Mean platelet volume is more important than age for defining reference intervals of platelet counts
Source: PLoS One. 2019 Mar 14;14(3):e0213658. doi: 10.1371/journal.pone.0213658 (PMC6417788; doi:10.1371/journal.pone.0213658)

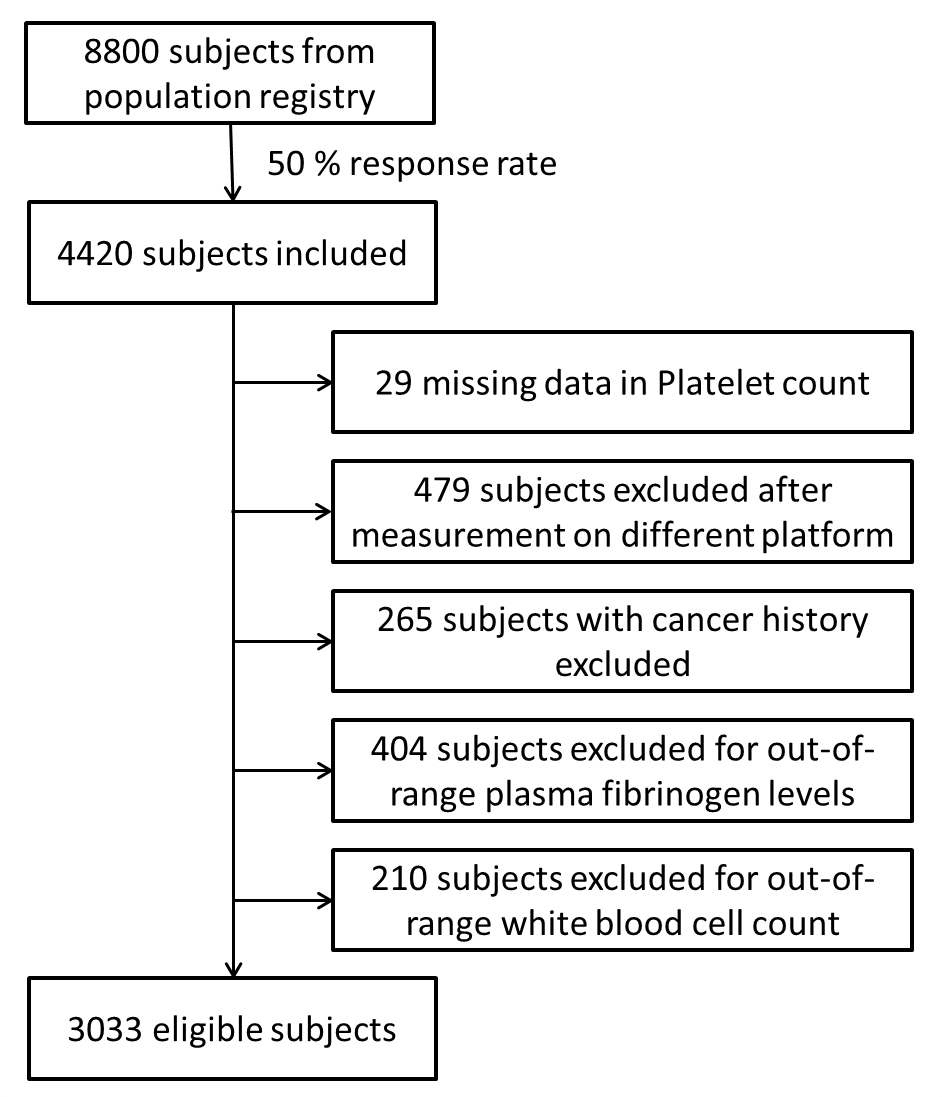

Supplement: S1 Fig — (TIF) [file pone.0213658.s003.tif]

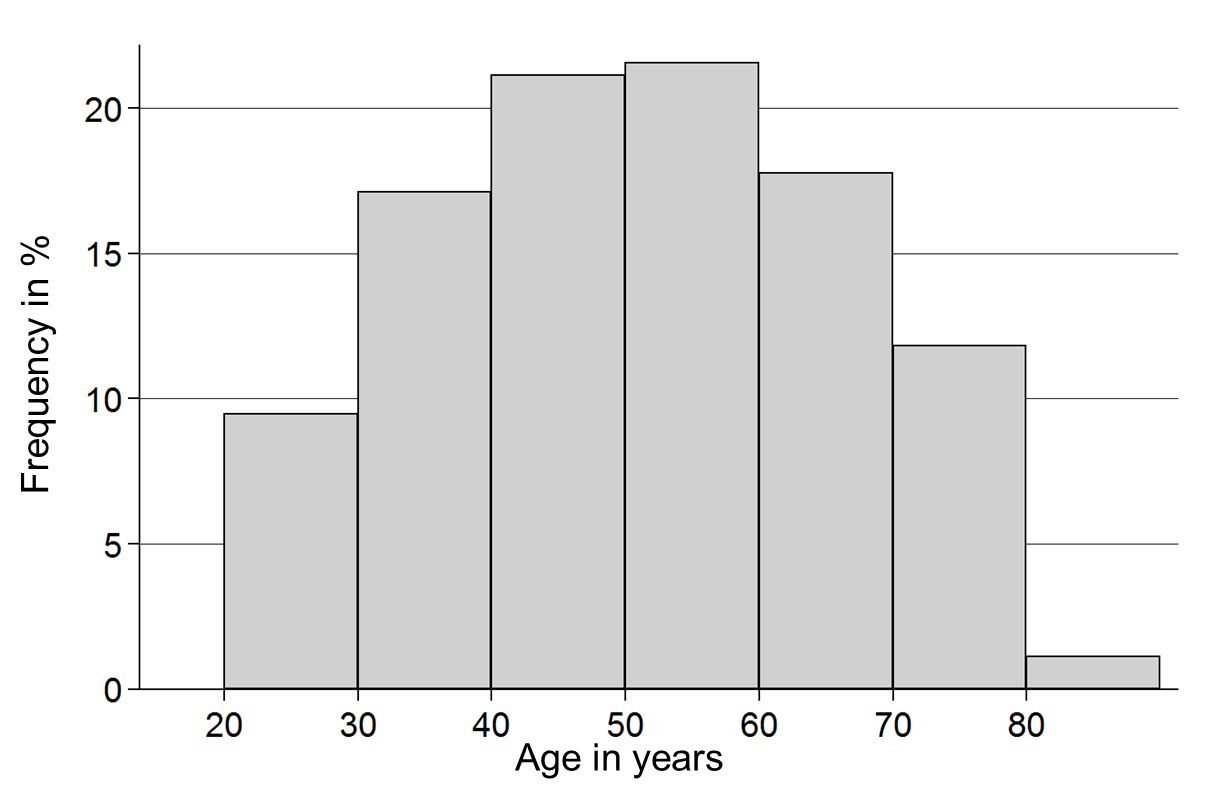

Supplement: S2 Fig — (TIF) [file pone.0213658.s004.tif]
